# Attenuation of oxidative stress in U937 cells by polyphenolic-rich bark fractions of *Burkea africana* and *Syzygium cordatum*

**DOI:** 10.1186/1472-6882-13-116

**Published:** 2013-05-28

**Authors:** Werner Cordier, Mary Gulumian, Allan Duncan Cromarty, Vanessa Steenkamp

**Affiliations:** 1 Department of Pharmacology, Faculty of Health Sciences, School of Medicine, University of Pretoria, P.O. Box X323, Arcadia, Pretoria, 0007, , South Africa; 2 Toxicology and Biochemistry Section, National Institute for Occupational Health, Johannesburg, South Africa; 3 Department of Haematology and Molecular Medicine, Faculty of Health Sciences, University of Witwatersrand, Johannesburg, South Africa

**Keywords:** Antioxidant, Apoptosis, *Burkea africana*, Cytotoxicity, Free radicals, Glutathione, Lipid peroxidation, Oxidative stress, Polyphenols, *Syzygium cordatum*

## Abstract

**Background:**

Oxidative stress has been implicated in the progression of various diseases, which may result in the depletion of endogenous antioxidants. Exogenous supplementation with antioxidants could result in increased protection against oxidative stress. As concerns have been raised regarding synthetic antioxidant usage, the identification of alternative treatments is justified. The aim of the present study was to determine the antioxidant efficacy of *Burkea africana* and *Syzygium cordatum* bark extracts in an *in vitro* oxidative stress model.

**Methods:**

Cytotoxicity of crude aqueous and methanolic extracts, as well as polyphenolic-rich fractions, was determined in C2C12 myoblasts, 3T3-L1 pre-adipocytes, normal human dermal fibroblasts and U937 macrophage-like cells using the neutral red uptake assay. Polyphenolic content was determined using the Folin-Ciocalteau and aluminium trichloride assays, and antioxidant activity using the Trolox Equivalence Antioxidant Capacity and DPPH assays. The extracts efficacy against oxidative stress in AAPH-exposed U937 cells was assessed with regards to reactive oxygen species generation, cytotoxicity, apoptosis, lipid peroxidation and reduced glutathione depletion.

**Results:**

*B*. *africana* and *S*. *cordatum* showed enrichment of polyphenols from the aqueous extract, to methanolic extract, to polyphenolic-rich fractions. Antioxidant activity followed the same trend, which correlated well with the increased concentration of polyphenols, and was between two- to three-fold stronger than the Trolox antioxidant control. Both plants had superior activity compared to ascorbic acid in the DPPH assay. Polyphenolic-rich fractions were most toxic to the 3T3-L1 (IC_50_’s between 13 and 21 μg/ml) and C2C12 (IC_50_’s approximately 25 μg/ml) cell lines, but were not cytotoxic in the U937 and normal human dermal fibroblasts cultures. Free radical-induced generation of reactive oxygen species (up to 80%), cytotoxicity (up to 20%), lipid peroxidation (up to 200%) and apoptosis (up to 60%) was successfully reduced by crude extracts of *B*. *africana* and the polyphenolic-rich fractions of both plants. The crude extracts of *S*. *cordatum* were not as effective in reducing cytotoxic parameters.

**Conclusion:**

Although oxidative stress was attenuated in U937 cells, cytotoxicity was observed in the 3T3-L1 and C2C12 cell lines. Further isolation and purification of polyphenolic-fractions could increase the potential use of these extracts as supplements by decreasing cytotoxicity and maintaining antioxidant quality.

## Background

Worldwide there is a high burden of disease, including diabetes mellitus, cardiomyopathy, nephropathy, cancer and neurodegenerative disorders [1,2]. Although reactive oxygen species (ROS) are formed during normal cellular functioning, high concentrations result in a period of oxidative stress [1]. During oxidative stress the human body is not able to maintain a homeostasis between endogenous antioxidants and ROS [1-3], with subsequent oxidation-induced damage to biomolecules. Damage incurred may present itself as apoptosis, lipid peroxidation, DNA degradation, protein modification, inflammation and ultimately cellular death [3], which may aggravate oxidative stress-related disorders [4].

Antioxidants are molecules that decrease the propagation and activity of free radicals through neutralization and quenching reactions [2]. Endogenous antioxidants, including enzymes (such as superoxide dismutase) and molecules (such as reduced glutathione [GSH]), aim to maintain homeostasis and reduce the deleterious effects of oxidative stress [2,3]. GSH is a non-protein thiol present at high levels in healthy cells, while the oxidized form (GSSG) appears at insignificant concentrations [5]. High concentrations of ROS can result in the depletion of GSH. During such periods the need for exogenous antioxidants becomes apparent. Due to controversies surrounding the potential cytotoxicity and carcinogenicity of synthetic antioxidants, novel antioxidant sources such as herbal remedies are therefore actively being investigated [6].

Plants are known to be rich antioxidant sources [7,8]. *Burkea africana* Hook.F (Fabaceae) and *Syzygium cordatum* Hochst. Ex C. Krauss (Myrtaceae) are two African plants that have been described in literature to contain high levels of polyphenols and antioxidants in their bark extracts [7,8]. The former, otherwise commonly known as the wild seringa, is used to treat heavy menstruation, abdominal pain, inflammation and pneumonia [7], while the latter, otherwise known as the waterberry, is used ethnomedicinally as an emetic, treatment of diarrhea, stomach aches and chest complaints [8].

The aim of the present study was therefore to investigate the polyphenolic content of the bark extracts of *B*. *africana* and *S*. *cordatum*, assess their antioxidant activity and/or cytotoxicity, as well as their efficacy to protect against oxidative stress in an *in vitro* cellular model, with the hope of determining their suitability of use as supplementary antioxidants.

## Methods

### Plant material and extract preparation

#### Plant material

Bark of *B*. *africana* and *S*. *cordatum* were collected during Spring (September) by Mr Lawrence Tshikhudo in Venda and Dr N Hahn in Machado (Limpopo), respectively. The identity of the plants was confirmed by Dr Hahn and voucher specimens deposited at the Department of Toxicology (*B*. *africana*, LT15) (Onderstepoort Veterinary Institute, Pretoria, South Africa) and the Soutpansbergensis Herbarium, Makado (*S*. *cordatum*, NH1880). Bark was cleaned, air-dried and ground to a fine powder (YellowLine Grinder, Merck).

#### Preparation of crude extracts

Bark powder (50 g) was macerated in 500 ml distilled water or methanol, sonicated for 30 min and kept at 4˚C for 24 h. The supernatant was stored, and the marc extracted for a second time following the same method. The respective extracts were combined and vacuum-filtered (0.22 μm). Aqueous and methanolic extracts were concentrated through *in vacuo* lyophilization (Freezone® 6 Freeze Dry System, Labconco) or rotary-evaporation (Büchi Rotovapor R-200, Büchi), respectively. Methanolic extracts were reconstituted in absolute ethanol to a 100 mg/ml stock. All extracts were dissolved prior to use to the desired concentrations (serial dilutions from 1 mg/ml) in phosphate buffered saline (PBS) and stored at -20˚C.

#### Preparation of polyphenolic-rich fractions

Polyphenolic-rich fractions were prepared according to the method of Jung *et al*. [9]. Bark powder (50 g) was defatted twice for 2 h with 80 ml hexane on a mechanical shaker. The hexane solvent was discarded, the defatted bark powder was air-dried and macerated in 200 ml methanol:acetone:water (80:15:5) at 4°C for 24 h. The extract was then vacuum-filtered (0.22 μm) and concentrated through *in vacuo* rotary-evaporation to 10 ml. Thereafter, the extract was mixed with 100 ml acidified water (pH 2, water phase) and subjected to liquid-liquid extraction (five times) for 1 h using 100 ml diethyl ether-ethyl acetate (1:1, DE/EA, organic phase). The organic phase was stored at -20°C until use.

The water phase was neutralized to pH 7 using 2 M sodium hydroxide, lyophilized and hydrolyzed with 100 ml 2 M sodium hydroxide for 4 h on a mechanical shaker at room temperature. The solution was then acidified to pH 2 with 6 M hydrochloric acid, and again subjected to liquid-liquid extraction as described above. The organic phases were combined, dehydrated with anhydrous sodium sulphate, vacuum-filtered (0.22 μm) and evaporated to dryness through *in vacuo* rotary-evaporation to form the polyphenolic-rich fraction. The evaporated fraction was dissolved in absolute ethanol to 100 mg/ml, dissolved to desired concentrations (serial dilutions from 1 mg/ml) in PBS and aliquots stored at -20˚C until use.

### Phytochemical screening

Phytochemical screening of crude extracts and polyphenolic-rich fractions for alkaloids, ascorbic acid, coumarins, specific flavonoids (apigenin, catechin, daidzein, epigallocatechin, genistein, hesperidin, kaempferol, myricetin, quercetin, rutin, sinapic acid and vitexin) and phenolic acids (benzoic acid, caffeic acid, ferulic acid, gallic acid, p-coumaric acidand syringic acid) were performed using thin-layer chromatography (TLC) [10]. The presence of glycosides, terpenoids and steroids was determined using biochemical reactions [11]. Glycoside presence was identified by a red-brown reaction upon treatment with sulphuric acid and ferric chloride. Terpenoid and steroid presence was determined using sulphuric acid, where a red-violet and green-blue reaction was a positive indication, respectively.

### Determination of total polyphenolic content (phenolic acids and flavonoids)

#### Total phenolic content (TPC)

The TPC of the crude extracts and polyphenolic-rich fractions was determined using the Folin-Ciocalteu assay as described by Slinkard & Singleton [12]. A standard curve was prepared using gallic acid. Into a tube was pipetted: 75 μl gallic acid standards (half serial dilutions of 1 mg/ml), crude extract, or polyphenolic-rich fraction, as well as 5925 μl distilled water and 375 μl Folin-Ciocalteu reagent. Tubes were incubated for 8 min after which 1125 μl sodium carbonate solution (20%) was added. Tubes were agitated and incubated in the dark for 2 h. Absorbance was measured at 765 nm (Lambda 25 UV/VIS Spectrophotometer, Perkin Elmer). Results are expressed as gallic acid equivalents (GAE mg/g extract ± SEM) which were calculated using the following equation:

$$\mathrm{GAE}=\frac{c\times v\times \mathrm{DF}}{m}$$

where, c = concentration calculated from standard curve (in mg/ml); v = volume obtained from initial extraction of plant material (in ml); DF = dilution factor of sample; and m = total weight of extract (in g).

#### Total flavonoid content (TFC)

The TFC of the crude extracts and polyphenolic-rich fractions was determined using the aluminium trichloride assay as described by Dewanto *et al*. [13]. A standard curve was prepared using rutin hydrate. Into a 96-well plate was pipetted: 20 μl rutin hydrate standard (half serial dilutions of 1 mg/ml), crude extract, or polyphenolic-rich fraction, as well as 20 μl sodium nitrate solution (3%), 20 μl aluminum trichloride solution (1%) and 100 μl sodium hydroxide solution (0.5 M). Absorbance was measured at 570 nm (ELx800 Universal Microplate Reader, Bio-Tek Instruments, Inc.). Results are expressed as rutin equivalents (RE mg/g extract ± SEM) which were calculated using the following equation:

$$\mathrm{RE}=\frac{c\times v\times \mathrm{DF}}{m}$$

where, c = concentration calculated from standard curve (in mg/ml); v = volume obtained from initial extraction of plant material (in ml); DF = dilution factor of sample; and m = total weight of extract (in g).

### Determination of cell-free antioxidant activity

#### Trolox equivalence antioxidant capacity (TEAC) assay

The 2,2’-azino-bis-(3-ethyl benzothiazoline 6-sulfonic acid) radical ABTS^•+^ scavenging activity of crude extracts and polyphenolic-rich fractions were determined using the TEAC assay as described by Re *et al*. [14]. Aqueous ABTS^•+^ (7.46 mM) was prepared in distilled water and oxidized using 2.5 mM potassium peroxidisulfate at 4°C for 16 h. ABTS^•+^ was diluted with distilled water to an absorbance of 0.70 ± 0.02 absorbance units at 734 nm. A standard curve was prepared using Trolox (half serial dilutions of 1 mg/ml) and samples were tested at four different concentrations (0.05 to 0.6 mg/ml). Into a cuvette was pipetted: 20 μl Trolox standard, crude extract, polyphenolic-rich fraction or ascorbic acid (as antioxidant control) as well as 2 ml ABTS^•+^. Absorbance was measured at 734 nm after 1 min incubation. Results are expressed as Trolox equivalents (TE ratio ± SEM) which were calculated using the following equation:

$$\mathrm{TE}=\frac{\mathrm{slope}\left(T\right)}{\mathrm{slope}\left(S\right)}$$

where, slope(T) = slope of Trolox standards curve; slope(S) = slope of sample curve.

#### 1,1-diphenyl-2-picrylhydrazyl (DPPH) radical assay

The DPPH radical scavenging activity of crude extracts and polyphenolic-rich fractions were determined using the DPPH assay as described by Gyamfi *et al*. [15]. A standard curve was prepared using Trolox (half serial dilutions of 0.5 mg/ml) and samples were tested at four different concentrations (0.05 to 0.6 mg/ml). Into a 96-well plate was pipetted: 15 μl Trolox standard, crude extract, polyphenolic-rich fraction or ascorbic acid as well as 185 μl DPPH solution (240 μM). Absorbance was measured after 15 min at 570 nm. Results are expressed as TE (ratio ± SEM) using the equation in Section Trolox equivalence antioxidant capacity (TEAC) assay.

### Cytotoxicity

#### Culture, maintenance and seeding of cells

Normal human dermal fibroblasts (NHDF) were purchased from Southern Medical (South Africa), while 3T3-L1 murine pre-adipocyte (#CL-173) and C2C12 murine myoblast (#CRL-1722) cell lines were purchased from the American Type Culture Collection. Adherent NHDF, 3T3-L1 and C2C12 cells were cultured in 10% foetal calf serum-supplemented Dulbecco’s Modified Eagle Medium (DMEM) with penicillin (100 U/ml) and streptomycin (100 μg/ml) at 37˚C and 5% CO_2_. Once cells became confluent, flasks were rinsed with PBS and cells enzymatically detached with Trypsin/Versene solution for 5 to 10 min. Cells were washed (200 *g*, 5 min) (TJ-6 Centrifuge, Beckman) and resuspended to 5 × 10^4^ cells/ml in 2% FCS-supplemented DMEM after determination of viability using the trypan blue exclusion assay (MicroStar 110, Reichert-Jung) and a haemocytometer.

The U937 human pro-monocytic cell line was obtained from European Collection of Cell Cultures (Sigma). Non-adherent U937 cells were cultured in 10% FCS-supplemented Roswell Park Memorial Institution (RPMI)-1640 with penicillin (100 U/ml) and streptomycin (100 μg/ml) at 37˚C and 5% CO_2._ Cells were washed (200 *g*, 5 min), counted using the trypan blue exclusion assay and diluted to 1 × 10^6^ cells/ml in 10% FCS-supplemented RPMI-1640. Cells were differentiated for 48 h with 32 nM phorbol-12-myristate-13-acetate (PMA) at 37˚C and 5% CO_2_. Cells were harvested and recounted using the trypan blue exclusion assay after differentiation and diluted to 1 × 10^6^ cells/ml in 2% FCS-supplemented RPMI-1640.

Into a 96-well plate was pipetted: a 100 μl cell suspension (5 × 10^3^ cells/well and 1 × 10^5^ cells/well for adherent and non-adherent cells, respectively) and 80 μl 2% FCS-supplemented medium. Plates were incubated at 37˚C and 5% CO_2_ for 1 h or 24 h for non-adherent or adherent cell lines, respectively.

#### Cytotoxicity of crude extracts and polyphenolic-rich fractions

Cytotoxicity was determined using the neutral red uptake assay as described by Borenfreund *et al*. [16]. The final concentration of ethanol used in the cellular assays for the methanolic extract and polyphenolic-rich fraction did not exceed 0.1%. The cytotoxicity of crude extracts and polyphenolic rich samples was determined in pre-seeded plates by addition of 20 μl medium (negative control), crude extracts or polyphenolic-rich fractions (0.0078 mg/ml to 1 mg/ml) and incubation for 72 h at 37˚C and 5% CO_2_. Medium was replaced with 100 μl neutral red medium (200 μg/ml) and incubated for 3 h after which plates were washed with PBS (200 *g*, 5 min). Plates were left to dry, the dye dissolved using 100 μl neutral red eluent (ethanol:distilled water:acetic acid [49:50:1]) and the absorbance measured at 540 nm (and blanked to the reference wavelength, 630 nm).

### Attenuation of oxidative stress-induced parameters in U937 cells

The oxidant *2*,*2*’-*azobis*-*(2-methylpropionamidine) dihydrochloride* (AAPH) is able to generate free radicals such as hydroxyls during thermolysis reactions [17,18]. During generation of ROS, cells undergo cytotoxicity that can be detected as GSH depletion, apoptosis and lipid peroxidation. These parameters can be measured using fluorometric and spectrophotometric assays.

#### Induction of AAPH-induced oxidative stress

Into pre-seeded U937 plates was pipetted: 20 μl medium (negative control), positive control (see respective sections), crude extract, polyphenolic-rich fraction (25, 50, 100 and 200 μg/ml) or 10 mM Trolox (as antioxidant comparison) and incubated for 1 h at 37°C and 5% CO_2_. In all experiments, except the ROS generation assays described in Section Efficacy to protect against AAPH-induced ROS generation, plates were washed with RPMI-1640 (200 *g*, 5 min), treated with 1.5 mM AAPH (final reaction volume 200 μl) and incubated for 48 h at 37˚C and 5% CO_2_.

All values were adjusted by subtraction of the blank. The results for percentage viability, apoptosis, lipid peroxidation and GSH depletion were expressed relative to the negative control using the following equation:

$$\mathrm{Parameter}\left(\%\right)=\frac{A\left(\mathrm{sample}\right)}{A\left(\mathrm{control}\right)}\times 100$$

where, A(control) = intensity of triplicate negative control; A(sample) = triplicate intensity of sample at a given concentration.

#### Efficacy to protect against AAPH-induced cytotoxicity

The ability of crude extracts and polyphenolic-rich fractions to attenuate AAPH-induced cytotoxicity in pre-seeded U937 plates was measured using the neutral red uptake assay as described in Section Cytotoxicity of crude extracts and polyphenolic-rich fractions. Saponin (1%) was used as positive control.

#### Efficacy to protect against AAPH-induced ROS generation

The ability of crude extracts and polyphenolic-rich fractions to attenuate AAPH-induced ROS generation was measured using the 2’, 7’-dichlorodihydrofluorescein diacetate (DCFHDA) method as described by Jakubowski and Bartosz [19]. Into pre-seeded U937 white plates was pipetted: a 20 μl medium, crude extract, polyphenolic-rich fraction (2.5, 5, 10 and 20 μg/ml) or 1 mM Trolox and 5 μM DCFHDA, which was incubated for 1 h at 37˚C and 5% CO_2_. Plates were washed with PBS (200 *g*, 5 min) and treated with 1.5 mM AAPH (final reaction volume 200 μl). Fluorescence was measured over a period of 3 h at λ_ex_ = 485 nm and λ_em_ = 520 nm (FLUOstar Optima, BMG Labtech). Percentage inhibition was determined using the following equation:

$$\mathrm{Inhibition}\left(\%\right)=\frac{\mathrm{AUC}\left(\mathrm{AAPH}\right)-\mathrm{AUC}\left(\mathrm{sample}\right)}{\mathrm{AUC}\left(\mathrm{AAPH}\right)}\times 100$$

where, AUC(AAPH) = average area under curve of AAPH-exposed cells; AUC(sample) = average area under curve of sample-treated, AAPH-exposed cells.

#### Efficacy to protect against AAPH-induced apoptosis

The ability of crude extracts and polyphenolic-rich fractions to attenuate AAPH-induced apoptosis was measured using the caspase-3 activity assay as described by Banjerdpongchai *et al*. [20]. Staurosporine (20 μM) was employed as positive control. Pre-seeded U937 AAPH-exposed plates were centrifuged (200 *g*, 5 min), medium replaced with 25 μl cold lysis buffer and incubated for 15 min on ice. Thereafter, a 100 μl caspase-3 substrate buffer containing Ac-DEVD-AMC was added and plates were incubated for 3 h at 37˚C. Fluorescence was measured at λ_ex_ = 355 nm and λ_em_ = 460 nm.

#### Efficacy to protect against AAPH-induced lipid peroxidation

The ability of crude extracts and polyphenolic-rich fractions to attenuate AAPH-induced lipid peroxidation was measured using the thiobarbituric acid (TBA) assay as described by Stern *et al*. [21]. Hydrogen peroxide (H_2_O_2_) (20 μM) was used as positive control. From pre-seeded U937 AAPH-exposed plates were taken aliquots of supernatant (150 μl), which were mixed with 200 μl trichloroacetic acid (12.5%) and 400 μl TBA (1%) and incubated at 95˚C for 20 min. 3-Methyl butan-1-ol (1 ml) was added to the mixture, vortex mixed and the organic layer was left to separate from the aqueous layer. Into a white 96-well plate was transferred 100 μl of the organic layer and the fluorescence measured at λ_ex_ = 544 nm and λ_em_ = 590 nm.

#### Efficacy to protect against AAPH-induced GSH depletion

The ability of crude extracts and polyphenolic-rich fractions to attenuate AAPH-induced GSH depletion was measured using the monochlorobimane assay as described by Fernandez-Checa and Kaplowitz [22]. H_2_O_2_ (20 μM) was used as positive control. Into pre-seeded U937 AAPH-exposed plates was pipetted 50 μM monochlorobimane and plates were incubated for 1 h. Plates were washed twice with PBS (200 *g*, 5 min) after which the fluorescence was measured at λ_ex_ = 355 nm and λ_em_ = 460 nm.

### Statistical analyses

All experiments were performed in triplicate on three separate days and results expressed as mean ± SEM using GraphPad Prism 4. The concentration needed to inhibit 50% of cell viability (IC_50_) was determined through using non-linear regression (variable slope). Results of attenuation of AAPH-induced parameters were compared with each other using one way analysis of variance (ANOVA) with a post-hoc Dunnett’s test. Significance was noted as p < 0.05.

## Results and discussion

### Phytochemical composition

In the present study alkaloids, flavonoids, glycosides, phenolic acids and terpenoids were identified in all the samples. Coumarins were only detected in the polyphenolic-rich fraction of *S. cordatum*. In the present study, polyphenols such as caffeic acid, cinnamic acid, epigallocatechin, gallic acid and sinapic acid were identified in both plants, while *B. africana* additionally tested positive for catechin and myricetin, and only *S. cordatum* for hesperidin. Flavonoids, glycosides, phenolic acids,proanthocyanidins, terpenoids and specific compounds such as catechin, epicatechin and fisetinidol have been described for the bark of *B. africana* in literature [7,23,24]. Compounds previously identified in the bark of *S. cordatum* include: alkaloids, flavonoids, phenolic acids (such as gallic acid), glucose, proanthocyanidins, a gallic-acid-ellagic acid complex, the leucoanthocyanidins (leucodelphinidin and leucocyanidin), a phytosterol (β-sitosterol) and the terpenoids arjunolic acid, friedelin and epi-friedelinol [25-27].

### Polyphenolic content and antioxidant activity

Crude extracts and the polyphenolic-rich fraction of *B. africana* contained approximately 2-fold higher polyphenolic content than *S. cordatum* (Table 1). Polyphenolic content increased by approximately 1.8-fold from the aqueous to methanolic extracts in both plants. A further 1.4-fold and 2-fold increase was seen from methanolic extracts to polyphenolic-rich fractions for *B. africana* and *S. cordatum*, respectively.

**Table 1:** Total polyphenolic content and antioxidant activity of crude extracts and polyphenolic-rich fractions

**Plant**	**Extract/Fraction**	**TPC (GAE mg/g extract ± SEM)**	**TFC (RE mg/g extract ± SEM)**	**Antioxidant activity**	
				**(TE ± SEM)**	
				**TEAC**	**DPPH**
*B. africana*	Aqueous	260.1 ± 10.8	196.8 ± 7.3	1.2 ± 0.0	1.1 ± 0.0
	Methanolic	484.4 ± 13.7	334.3 ± 3.0	1.4 ± 0.0	1.5 ± 0.0
	Polyphenolic	699.1 ± 25.3	460.0 ± 18.9	2.0 ± 0.1	2.2 ± 0.0
*S. cordatum*	Aqueous	183.9 ± 5.6	130.6 ± 9.5	0.8 ± 0.0	0.7 ± 0.0
	Methanolic	260.8 ± 9.2	192.2 ± 8.9	1.1 ± 0.0	1.1 ± 0.0
	Polyphenolic	619.4 ± 11.3	334.0 ± 9.7	2.5 ± 0.1	3.0 ± 0.1
Ascorbic acid		-	-	2.8 ± 0.0	1.7 ± 0.0

*GAE*, Gallic acid equivalents; *TFC*, Total flavonoid content; *RE*, Rutin equivalents; *TE*, Trolox equivalents.

The antioxidant activity of the crude extracts of *B. africana* was higher than that of *S. cordatum*, whereas the opposite was seen for the polyphenolic-rich fractions (Table 1). Antioxidant activity could be explained by the presence of polyphenols as a correlation was seen: phenolic content, r > 0.85; flavonoid content, r > 0.72. The antioxidant activity was similar between the TEAC and DPPH assays, which could be explained due to the similar mechanism of quenching in both assays. Antioxidant activity of *B. africana* has been reported using the β-carotene [23] and the DPPH radical scavenging assays [7,23]. Previously, antioxidant activity of crude extracts of *S. cordatum* bark was reported to be higher in the aqueous extract (1.95 TE) than the methanolic extract (0.80 TE) [27]. Hydroxyl groups are known to increase radical scavenging activity [28]. Leucocyanidin, leucodelphinidin, catechin, epicatechin, myricetin and proanthocyanidins (all of which have been described in both plants) contain numerous hydroxyl groups. Importantly, both plants had superior activity to ascorbic acid in the DPPH assay, indicating more potent free radical scavenging ability than this known commercial antioxidant supplement.

### Cytotoxicity of crude extracts and polyphenolic-rich fractions

Literature could not be obtained for comparison with regards to cytotoxicity testing using the cell lines in the present study. Crude extracts and polyphenolic-rich fractions displayed cytotoxicity in the C2C12 and 3T3-L1 cell lines, but not in the U937 and NHDF cells (Table 2). The highest cytotoxicity was observed for the polyphenolic-rich fractions. The methanolic extract and polyphenolic-rich fraction of *B. africana* were more cytotoxic than the aqueous extract, whereas the methanolic extract of *S. cordatum* was less toxic than its aqueous extract. The C2C12 cell line was more susceptible to the cytotoxicity induced by *B. africana* extracts than the 3T3-L1 cell line, while the inverse was observed with *S. cordatum* extracts.

**Table 2:** The cytotoxicity of crude extracts and polyphenolic-rich fractions on different cell line cultures

**Plant**	**Extract/Fraction**	**IC**_**50**_ **± SEM (μg/ml)**			
		**C2C12**	**3T3-L1**	**NHDF**	**U937**
*B. africana*	Aqueous	42.5 ± 1.1	84.5 ± 1.0	>100	>100
	Methanolic	16.0 ± 1.0	28.5 ± 1.0	>100	>100
	Polyphenolic	13.9 ± 1.0	24.3 ± 1.0	>100	>100
*S. cordatum*	Aqueous	63.4 ± 1.0	31.4 ± 1.0	>100	>100
	Methanolic	95.6 ± 1.1	74.6 ± 1.0	>100	>100
	Polyphenolic	20.5 ± 1.1	25.0 ± 1.0	>100	>100

In the present study, the cytotoxicity of the aqueous extract of *B. africana* (84.5 μg/ml) in 3T3-L1 cells was comparable to that described for an ethanolic stem extract using the brine shrimp toxicity assay (87.24 μg/ml).[29] The cytotoxicity noted for the crude extracts and polyphenolic-rich fraction of *S. cordatum* is comparable to that obtained for dichloromethane:methanol and acetone extracts using human kidney epithelial cells [30] and green monkey Vero cells [31], although the latter cells showed no toxicity to methanolic extracts [31,32].

Although the mechanism of action of cytotoxicity of the crude extracts and polyphenolic-rich fractions observed in the present study and in those reported in the literature is not known, indications are that polyphenols may produce cell death in the 3T3-L1 cells due to their inherent anti-adipogenic activity [33], by inducing cell cycle disturbances and initiation of apoptosis [33,34].

### Attenuation of oxidative stress-induced parameters in U937 cells

#### Protection against AAPH-induced ROS generation, lipid peroxidation, apoptosis and cytotoxicity

**Figure 1:**
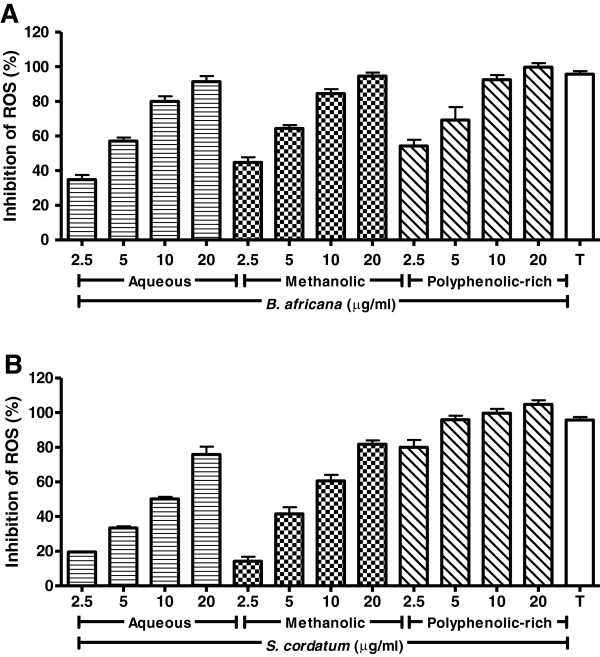
**Inhibition of AAPH-induced ROS generation in U937 cells pre-treated with crude extracts and polyphenolic-rich fractions.** Legend: **A**) *B. africana* and **B**) *S. cordatum*.

AAPH generated approximately 17-fold higher levels of ROS than the negative control over a 3 h period. Pre-treatment with crude extracts and polyphenolic-rich fractions for 1 h reduced this ROS generation (Figure 1). While crude extracts of *B. africana* had superior activity compared to that of *S. cordatum*, the polyphenolic-rich fraction of *S. cordatum* surpassed that of *B.* africana, resulting in 80% inhibition at 2.5 μg/ml.

**Figure 2:**
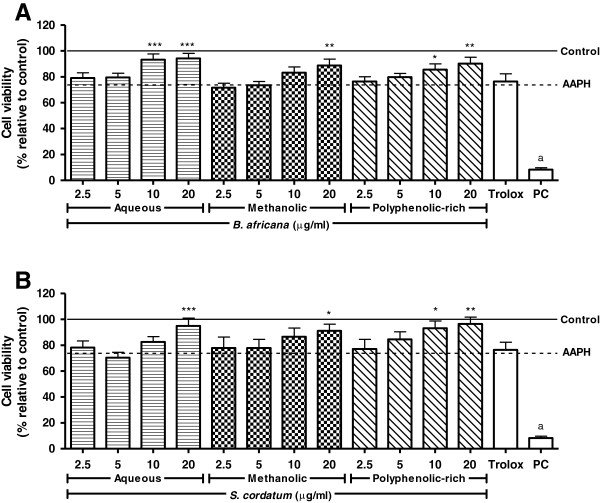
**Protection against AAPH-induced cytotoxicity in U937 cells pre-treated with crude extracts and polyphenolic-rich fractions.** Legend: **A**) *B. africana* and **B**) *S. cordatum*; PC = Positive control (saponin); solid and dashed lines represent negative control and AAPH-treated cells, respectively; control vs AAPH: a – p < 0.001; AAPH vs sample: *** – p < 0.001, ** – p < 0.01, * – p < 0.05.

Cell viability was reduced to 73.68% after exposure to AAPH, but pre-treatment with crude extracts and polyphenolic-rich fractions (10 and 20 μg/ml) of both plants increased survival of cells to above 80% (Figure 2). The polyphenolic-rich fraction of *S. cordatum* tended to protect the cells to a greater degree than *B. africana* but the difference in this protection was not significant.

**Figure 3:**
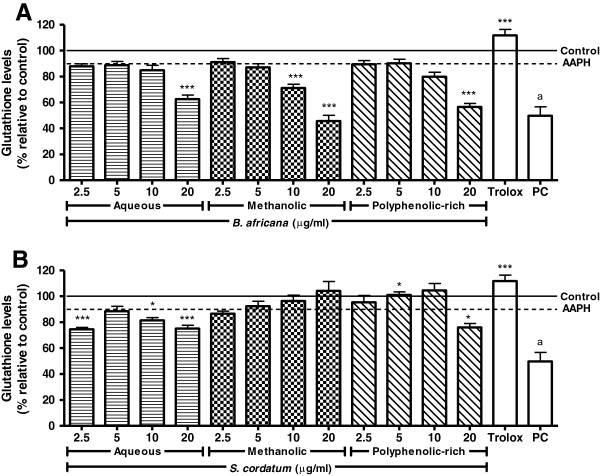
**Inhibition of AAPH-induced lipid peroxidation in U937 cells pre-treated with crude extracts and polyphenolic-rich fractions.** Legend: **A**) *B. africana* and **B**) *S. cordatum*; PC = Positive control (H_2_O_2_); solid and dashed lines represent negative control and AAPH-treated cells, respectively; control vs AAPH: a – p < 0.001; AAPH vs sample: *** – p < 0.001, ** – p < 0.01, * – p < 0.05.

Lipid peroxidation was induced after exposure to AAPH as measured by MDA formation (384.4%) (Figure 3). Reduction of MDA concentrations was observed for both crude extracts and polyphenolic rich fractions of *B. africana*. However, only the polyphenolic-rich fraction of *S. cordatum* had a protective effect.

**Figure 4:**
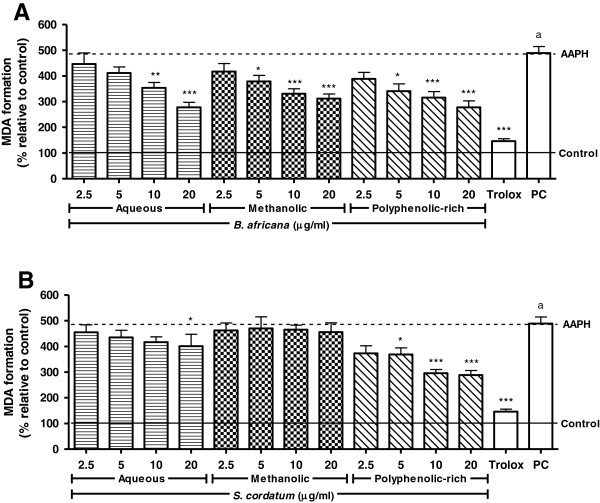
**Inhibition of AAPH-induced caspase-3 activation in U937 cells pre-treated with crude extracts and polyphenolic-rich fractions.** Legend: **A**) *B. africana* and **B**) *S. cordatum*; PC = Positive control (staurosporine); solid and dashed lines represent negative control and AAPH-treated cells, respectively; control vs AAPH: a – p < 0.001; AAPH vs sample: *** – p < 0.001, ** – p < 0.01, * – p < 0.05.

AAPH-exposure increased apoptosis as measured by caspase-3 activity by 33.6% (Figure 4). While all samples of *B. africana* were able to decrease caspase-3 activity, only the polyphenolic-rich fraction of *S. cordatum* elicited a definitive response. Apoptosis was decreased significantly (*p* < 0.05) in a dose-dependent manner by *B. africana*, with the polyphenolic-rich fraction being most potent, followed by the methanolic and aqueous extracts. The crude extracts of *S. cordatum* were less cytoprotective and did not follow a specific trend. All concentrations of the polyphenolic-rich fraction of *S. cordatum* decreased caspase-3 activity significantly (*p* < 0.05).

All samples of *B. africana* and the polyphenolic-rich fraction of *S. cordatum* resulted in protection against lipid peroxidation and apoptosis. A larger decrease in apoptosis was seen in the polyphenolic-rich fraction of *B. africana* than that of *S. cordatum,* even though the latter had greater antioxidant activity. This could suggest the presence of non-antioxidant phytochemicals contributing to the anti-apoptotic effect. The lack of effect seen with the crude extracts of *S. cordatum* could be attributed to the lower polyphenolic content and antioxidant activity of these crude extracts (as seen in the cell-free antioxidant assays). Furthermore, cytoprotection is dependent on the amount of phytochemicals that are able to move across the cell membrane [35]. The inhibition of lipid peroxidation decreases the formation of peroxidation by-products, which have been implicated in the induction of pro-apoptotic factors [36]. Thus apoptosis could be reduced through inhibition of lipid peroxidation. Additionally, apoptosis could be deterred through mitochondrial stabilization, deactivation of caspase cascades or reversal of ROS generation [37].

Hydroethanol extracts of *B. africana* were reported to decrease bovine phospholipid damage (IC_50_ = 78 μg/ml) [7] and although no IC_50_ was obtained in the present study, a 43% inhibition of lipid peroxidation was seen for the polyphenolic-rich fraction at 20 μg/ml. Previously, reduced lipid peroxidation has been attributed to polyphenolic and anthocyanin content of *Syzygium cumini* Skeels [38]*.*

The possibility exists that polyphenols that are present within both plants (as described in the literature or identified in the present study using TLC) may have contributed to the decrease in oxidative stress parameters assessed in U937 cells. This may include caffeic acid [39], catechin, epicatechin [40] and gallic acid [41]. Myricetin, which was identified in *B. africana*, has been shown to reduce 1-methyl-4-phenyl-1,2,3,6-tetrahydropyridin-induced ROS generation and cytotoxicity in MES23.5 rat dopaminergic cells [42]. This antioxidant has also been reported to reduce phosphorylation of MKK4 and JNK, neutralize free radicals (all of which reverse mitochondrial dysfunction), apoptosis and DNA damage [42]. Anthocyanins [43], similar to the anthocyanidins leucocyanidin and leucodelphinidin, and the triterpenoid, arjunolic acid [44], have been shown to reduce oxidative stress *in vitro* which can contribute to the cytoprotective effects seen with *S. cordatum* pre-treatment by decreasing apoptosis and lipid peroxidation. Studies carried out on individual polyphenols and phytochemicals identified in the plants studied, such as arjunolic acid, catechin, epicatechin, gallic acid, hesperidin and myricetin reported decreased lipid peroxidation [44-47] and apoptosis [35,42,44,48,49] induced by oxidative stress. Isoflavones, present in most plants of the Fabaceae family, decrease oxidative-induced lipid damage through incorporation into low-density lipid particles [50], which might explain the reduction seen in all samples of *B. africana*. Procyanidins stabilize lipid membranes, which decreases the movement of ROS into the hydrophobic intracellular compartment and as such attenuates damage [51]. The most described mechanisms of reduced apoptosis were through the reduction in pro-apoptotic signals, upregulation of anti-apoptotic proteins and stabilization of the mitochondrial membrane. Ultimately cell viability was increased in all cells pre-treated with samples of both plants, indicating that different factors are at play which is decreasing the cytotoxicity induced by AAPH – including the induction of necrosis, DNA damage, inflammatory responses and mitochondrial dysfunction. These are typical parameters that should be targeted during treatment of oxidative stress-related disorders, and as such antioxidant supplementation from the bark of these plants could assist in limiting damage brought upon cells.

#### Protection against AAPH-induced GSH depletion

**Figure 5:**
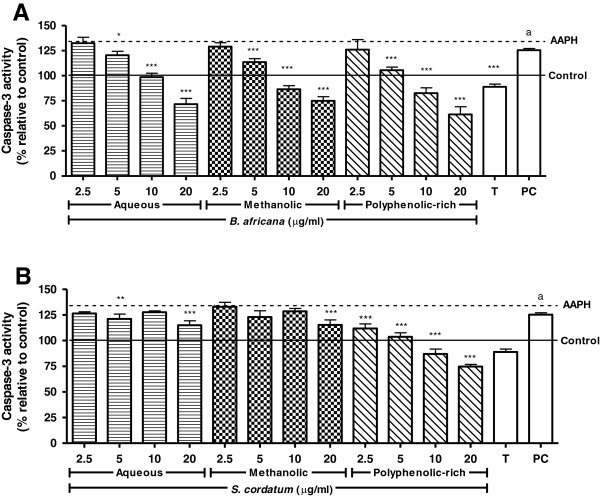
**Effect on AAPH-induced glutathione depletion in U937 cells pre-treated with crude extracts and polyphenolic-rich fractions.** Legend: **A**) *B. africana* and **B**) *S. cordatum*; PC = Positive control (H_2_O_2_); solid and dashed lines represent negative control and AAPH-treated cells, respectively; control vs AAPH: a – p < 0.001; AAPH vs sample: *** – p < 0.001, * – p < 0.05.

APPH exposure reduced GSH concentrations by 10.24% (Figure 5). All samples of *B. africana* resulted in a decrease in GSH concentrations, with the methanolic extract being the most potent. The aqueous extract of *S. cordatum* reduced GSH whereas the methanolic extract increased GSH concentrations (as expected from antioxidant supplementation).

The crude extracts and polyphenolic-rich fractions of *B. africana* as well as the aqueous extract of *S. cordatum* decreased intracellular GSH concentrations dose-dependently. Although studies support the increase of endogenous antioxidant stores brought about through antioxidant pre-treatment [52], this was not observed in the present study for *B. africana*.

Polyphenols such as flavonoids and catechins are reported to be able to decrease glutathione reductase activity [53], which in turn decreases the amount of GSH converted from GSSG. Furthermore, GSH-polyphenol complexes decrease the amount of GSH available to cells, by increasing GSSG/GSH ratio and ultimately being released extracellularly. Such conjugations can form from polyphenol *o-*quinone metabolites [54]. Although GSH depletion was noted, other antioxidant systems such as superoxide dismutase and catalase could have been affected and thus led to an increase in protection. Although the cells were essentially susceptible to oxidative attack due to decreased endogenous GSH, cell viability was not affected. The reduction of ROS brought on by pre-treatment with crude extracts and polyphenolic-rich fractions is thought to decrease the amount of free radicals available to damage cells. This however does not fully explain the protection in the absence of GSH, therefore stabilization of other parameters should be considered, such as decreased apoptotic factors and protection against lipid peroxidation.

## Conclusion

Although literature concerning *B. africana* and *S. cordatum* is limited, the bark of both of these plants is known to contain a variety of polyphenols, which justifies its potential use as an antioxidant supplement for oxidative stress-related disorders. Cytotoxic parameters such as cytotoxicity, ROS generation, lipid peroxidation and apoptosis were decreased by extracts from both plants studied. The highest activity was noted for the polyphenolic-rich fractions, which can largely be attributed to their higher polyphenolic content and antioxidant activities. Although reduction of oxidative stress-related parameters were observed, cytotoxicity was induced by extracts from both plants in the cultured 3T3-L1 and C2C12 cell lines. This could result in difficulties in development of an antioxidant supplementation. Further isolation and purification of polyphenolic-rich fractions could lead to identification and elimination of cytotoxic elements, while maintaining antioxidant potential. Such refinement could increase the potential of the polyphenolic-rich fraction to be used as a health-supplement.

## Competing interests

The authors declared that they have no competing interest.

## Authors’ contributions

WC conducted all experiments and drafted the manuscript. MG, ADC and VS provided critical appraisal of technical work and the manuscript. All authors read and approved the final manuscript.

## Pre-publication history

The pre-publication history for this paper can be accessed here:

http://www.biomedcentral.com/1472-6882/13/116/prepub
